# CAV1 Promotes HCC Cell Progression and Metastasis through Wnt/β-Catenin Pathway

**DOI:** 10.1371/journal.pone.0106451

**Published:** 2014-09-02

**Authors:** Hongxiu Yu, Huali Shen, Yang Zhang, Fan Zhong, Yinkun Liu, Lunxiu Qin, Pengyuan Yang

**Affiliations:** 1 Institutes of Biomedical Sciences of Shanghai Medical School, Fudan University, Shanghai, P. R. China; 2 Zhongshan Hospital, Fudan University, Shanghai, P. R. China; 3 Department of Chemistry, Fudan University, Shanghai, P. R. China; The University of Hong Kong, Hong Kong

## Abstract

Caveolin-1 (CAV1) has significant roles in many primary tumors and metastasis, despite the fact that malignant cells from different cancer types have different profiles of CAV1 expression. There is little information concerning CAV1 expression and role in hepatocellular carcinoma (HCC) progresion and metastasis. The role of CAV1 in HCC progression was explored in this study. We reported that CAV1 was overexpressed in highly invasive HCC cell lines compared with poorly invasive ones. The immunohistochemical staining was obviously stronger in metastatic HCC samples than in the non-metastatic specimens via tissue microarrays. Furthermore, CAV1 overexpression enhanced HCC cell invasiveness *in vitro*, and promoted tumorigenicity and lung metastasis *in vivo*. By contrast, CAV1 stable knockdown markedly reduced these malignant behaviors. Importantly, we found that CAV1 could induce EMT process through Wnt/β-catenin pathway to promote HCC metastasis. We also identify MMP-7 as a novel downstream target of CAV1. We have determined that CAV1 acts as a mediator between hyperactive ERK1/2 signaling and regulation of MMP-7 transcription. Together, these studies mechanistically show a previously unrecognized interplay between CAV1, EMT, ERK1/2 and MMP-7 that is likely significant in the progression of HCC toward metastasis.

## Introduction

Hepatocellular carcinoma (HCC) is one of the most common and aggressive human malignancies, and like other tumors, how HCC invade and spread remains a mysterious [1]. There are a number of genes found to play important roles in HCC malignance and metastasis, including Caveolin-1 (CAV1). CAV1 is the main component of caveolae membrane structures, and has been implicated in diverse human cancers. The function of CAV1 in cancer is reported to be cancer type- and stage-dependent [2], [3]. In HCC, stepwise increase in CAV1 expression during hepatocellular carcinogenesis has been reported [4]. Increased expression of CAV1 was associated with metastasis and with a worse prognosis of HCC [5], [6]. CAV1 expression correlates positively with vascular endothelial growth factor (VEGF), microvessel density (MVD) and unpaired artery (UA) [6], [7]. These results suggest that CAV1 may play an important role in the progression of HCC.

However, few reports gave some evidences that CAV1 could be considered as an tumor suppressor and anti-metastatic effector in HCC, for example, Yan et al reported that CAV1 expression is significantly decreased in hepatitis B virus (HBV)-infected HCC tissues and reversely correlates with HCC tumor progression [8]. Yang et al also reported that CAV1 may play a tumour-suppressive role in HCC [9]. CAV1's function as a tumor suppressor or promoter in HCC is still debated. CAV1 inhibits metastatic potential in melanomas through suppression of the integrin/Src/FAK signaling pathway in melanoma has been reported [10]. These observations do not provide clear evidence about the role of CAV1 in HCC progression.

In present study, we aimed to identify the expression status of CAV1 in HCC cell lines and tumor samples, in order to investigate the changes of CAV1 expression in HCC progression. CAV1 expression was examined in a panel of human HCC cell lines using western blotting analysis and quantitative RT-PCR and human tissues by immunohistochemistry. To investigate the functions of CAV1 in HCC metastasis, CAV1 overexpressing and knockdown stable clones were established in HCC cells. It's *in vitro* cellular effects and tumourigenicity and metastatic potential *in vivo* were examined. We complemented these descriptive analyses by studying its molecular mechanisms behind the increase in the motility and invasion.

## Materials and Methods

### Cell Culture

Cell-lines were cultured at 37°C in 5% CO_2_ in Dulbeccos modified Eagle medium (DMEM)(Gibco, U.S.A) supplemented with 10% fetal bovine serum (FBS, Life Technologies) in a 5% CO_2_-humidified chamber.

### Extraction of RNA and Quantitative Real-time RT-PCR

Total RNA was extracted using Trizol solution (Invitrogen) according to the protocols recommended by the manufacturer. RNAase-free DNase I was used to remove DNA contamination. The total RNA concentration and quantity were assessed by absorbance at 260 nm, using a DNA/Protein Analyzer (NanoDROP2000C; Thermo). Reverse transcription was performed on 5 µg of total RNA with an ologo(dT) primer.

To perform quantitative analysis of the expression of the CAV1 gene in cell lines, the relative mRNA level of CAV1 was measured by quantitative real-time RT-PCR, using the ABI 7500 Detection System and SYBR green dye (TaKaRa), according to the protocols recommended by the manufacturer. The following primers were used to amplify a 144-bp PCR product for CAV1: forward, 5′- GCAACATCTACAAGCCCA -3′; reverse, 5′- CTTCAAAGTCAA TCTTGACCAC-3′. The following primers were used to amplify E-cadherin: forward, 5′- GACCGGTGCAATCTTCAAA -3′; reverse, 5′-TTGACGCCGAGAGCTACAC-3′. The following primers were used to amplify Vimetin: forward, 5′-ATTCCAC TTTGCGTTCAAGG-3′; reverse, 5′-CTTCAGAGAGAGGAAGCCGA-3′. The following primers were used to amplify TWIST: forward, 5′- TCCATTTTCTC CTTCTCTGGAA -3′; reverse, 5′-GTCCGCGTCCCACTAGC -3′. A housekeeping gene, 18S RNA, was used as an endogenous control. The following primers were used to amplify 18S RNA: forward, 5′-CAGCCACCCGAGATTGAGCA-3′; reverse: 5′-TAGTAGCGACGGGCG GTGTG-3′. Measurements were repeated at least 3 times to ensure the reproducibility of the results.

### Preparation of stable CAV1-expressing cells

To construct CAV1 recombinant adenovirus vector, full length CAV1 cDNA was amplified by PCR from HCCLM3 cells cDNA. The primers were as follows: forward, 5′-GAATTCATGTCTGGGGGC-3′; reverse, 5′- GTCGACTTATATTTC TTTCTGC-3′. After digestion with *ECORI* and *Sal I*, the PCR product was inserted into the pBABE-Puro vector. After verified by sequencing, lentivirus production and transduction Virus was produced by triple transfection of pBABE-CAV1, VSVG and GAG into 293T cells using Lipofectamine 2000 (Invitrogen). Five hours after transfection, the medium was changed to DMEM containing 10% FBS. The medium was harvested 48 hours after transfection. HepG2 was infected by overnight incubation with medium containing the pBABE-CAV1 retrovirus and 8 ug/ml of Polybrene (Sigma). Stable pools were selected with 2 ug/ml puromycin (Sigma) for 7 days. Cells were stably transfected with pBABE-GFP as control.

### Construction and Transfection of Lentivial Vectors with Specific shRNAs for CAV1

For the construction of the RNAi plasmid, the oligonucleotides for the double-stranded shRNA were inserted into the expression plasmid pLKO.1 (Sigma). The oligonucleotides for shRNA were synthesized as follows: shRNA-1, forward, CCGGGACCCACTCTTTGAAGCTGTTCTCGAGAACAGCTTCAAAGAGTGGGTCTTTTTG; shRNA-1, reverse, AATTCAAAAAGACCCACTCTTTGAAGCTGTT CTCGAGAACAGCTTCAAAGAGTGGGTC, shRNA-2, forward, CCGGGACCCTAAACACCTCAACGATCTCGAGATCGTTGAGGTGTTTAGGGTCTTTTTG, shRNA-2, reverse, AATTCAAAAAGACCCTAAACACCTCAACGAT CTCGAGATCGTTGAGGTGTTTAGGGT for pLKO.1 CAV1; The negative control oligos served as the nonspecific control. To generate lentiviral particles, the desired plasmid and lentiviral packaging plasmids psPAX2 and pMD2. G (Sigma) were transfected into 293 T cells using FuGENE 6 (Roche, Germany). Medium was changed 24 hours after transfection and viral particles were harvested twice over the next 72 hours. Cells were infected with the lentiviral supernatant for 24 hours in the presence of 8 µg/ml of Polybrene (Sigma). Infected MHCC97-H and HCCLM3 cells were selected by addition of 5 µg/ml puromycin (Sigma).

### Tissue Microarray (TMA) Construction

TMA was constructed from 96 human HCCs to test CAV1 expression. The TMA contains 48 patients with metastatic HCC whose primary HCC lesions were accompanied by intrahepatic spreading, which had been regarded as the most frequently metastatic site of HCC, in portal vein, hepatic vein, or bile duct, and 48 patients who had only solitary HCC without metastases. Both staining intensity and percentage of positive cells were scored by two experienced pathologists. This TMA was constructed by a standard method from patients who underwent curative resection at the Liver Cancer Institute and Zhongshan Hospital (Fudan University, Shanghai, China). Ethical approval from Fudan University (Shanghai, PR China) Research Ethics Committee was obtained. The original tissues had been fixed in 4% buffered formalin and paraffin embedded according to routine procedures. New hematoxylin and eosin-stained slides from each case were performed for review and selection of the most representative areas. Briefly, for each sample, two 1 mm diameter cylinders of tissue were obtained from representative areas of each archived paraffin block and arrayed into a new recipient paraffin block with a custom-built precision instrument (Beecher Instruments, Silver Spring, MD, USA). After construction, initial sections of the TMA were stained for hematoxylin and eosin and reviewed by clinical pathologists to verify the histopathological findings.

### Immunohistochemistry Staining (IHC)

Immunohistochemical staining for CAV1 was carried out on sections of the formalin-fixed samples on the TMA. Briefly, the sections were deparaffinized in xylene and rehydrated by transfer through graded concentrations of ethanol to distilled water, and endogenous peroxidase activity was blocked by incubation with 30 mL/L H_2_O_2_ in methanol for 10 minutes at room temperature. Then, sections were submitted to antigen retrieval in a pressure cooker containing 0.01 mmol/L sodium citrate buffer for 10 minutes. Slides were subsequently incubated in 100 mL/L normal goat serum for 20 minutes at room temperature. Antibodies to CAV1 (SC-894, Santa Cruze) were incubated at 37°C for 2 hours and placed at 4°Covernight. Then, the slides were incubated with a horseradish peroxidase–conjugated mouse anti-rabbit secondary antibody (Genscript, P.R.China) at 37°C for 1 hour. Finally, the sections were reacted with 0.02% 3,3′-diaminoberzidine and 0.005% H_2_O_2_ in 0.05 mmol/L Tris-HCl buffer, and counterstaining was performed with hematoxylin. Negative control slides were treated by incubation with PBS instead of the primary mouse antibody. Stained slides were observed under light microscopy. All slides were reviewed independently by 2 pathologists who were blinded to each other's readings. CAV1 immunostaining were semi-quantitatively estimated based on proportion (percentage of positive cells). The proportion of caveolin-1 staining was classified into two categories: −(0∼25%), +(26∼100%) for statistical analysis. Antibodies to E-cadherin(610181) and Vimentin (ab92547) are purchased from BD Transduction Laboratories and Abcam respectively.

### *In vivo* metastasis assay via orthotopic implantation

We employed the orthotopic implantation assay to assess the effect of CAV1 on tumor metastasis. Mice were anesthetized with 2.5% sodium pentobarbital (40 mg/kg; Sigma), the right side of the abdomen was sterilely prepped. The abdomen was entered through a small opening in the muscle and peritoneum. Small piece of subcutaneous tumor was removed, minced to produce fresh tumor pieces about 1×1×1 mm^3^, and implanted into the liver of each of six nude mice. Then, the skin was closed. After 6 weeks, the mice were killed and autopsied.

The abdominal organs, including the liver, the lungs were sampled for standard histopathological studies as detailed above. Liver tumor wet weights were compared using the *t* test in SPSS (version 11.5; SPSS). Consecutive sections were made for every tissue block of the lung. Take first one of the every five consecutive sections for hematoxylin-eosin staining and examination for the lung metastasis. Based on the number of HCC cells in the maximal section of the metastatic lesion, the lung metastases were classified into four grades: grade I, ∼20 cells; grade II, 20–50 cells; grade III, 50–100 cells; and grade IV, ∼100 cells. This study was approved by the The Animal Care and Use Committee of Fudan University, China. All surgery was performed under sodium pentobarbital anesthesia, and all efforts were made to minimize suffering.

Experimental procedure for cell migration, wound-healing, anoikis, WB, reporter gene, and tumorigenicity are mentioned in Supplemental Experimental Procedures (File S1).

## Results

### CAV1 overexpression was necessary for HCC malignance and metastasis but has minor effect for HCC growth

**Figure 1:**
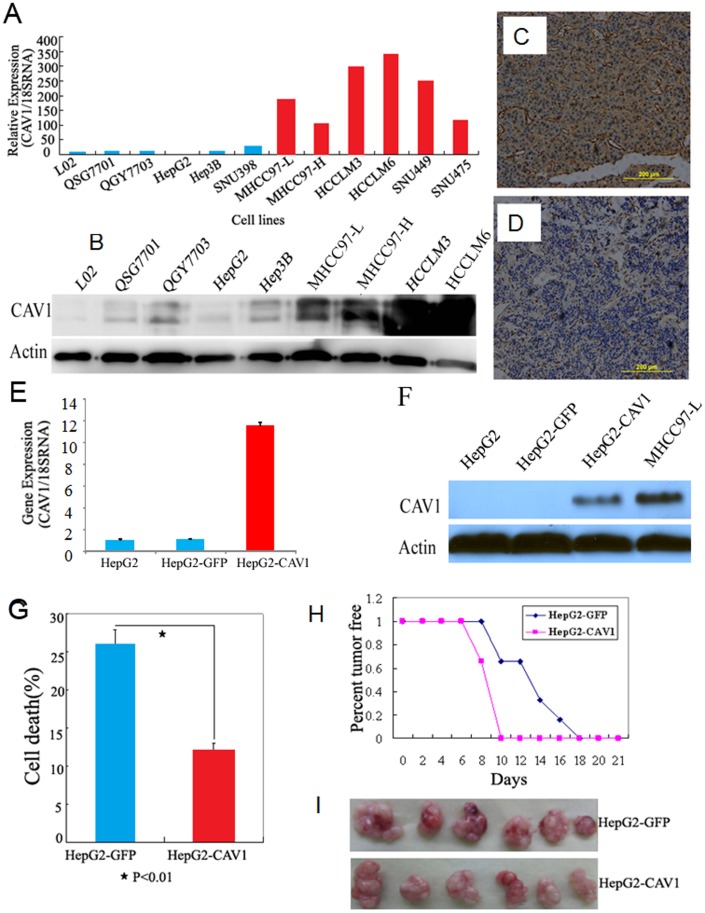
Expression levels of CAV1 in various HCC samples. 1A, 1B: CAV1 mRNA and protein levels of HCC cell lines detected by Q-PCR and Western Blotting. 1C, Membranous and strong cytoplasmic CAV1 expression in a metastatic HCC; 1D, Weak CAV1 expression in hepatocytes of a non-metastatic HCC and strong CAV1 expression in the endothelium of the blood vessels. Original magnification, ×200; 1E and 1F, Q-PCR and Western blot analysis indicated the expressed CAV1 in HepG2 cell lines. The CAV1 expression levels upon CAV1 adenovirus expression are comparable to the endogenous CAV1 level in MHCC97-L cell lines; 1G, Cell anoikis assays using poly-HEMA. Data are presented as mean values (n = 3); error bars represent ± SD; 4H, Stable CAV1 overexpression pools from HepG2 cells were injected subcutaneously into mice; each group contained 6 mice. A Kaplan-Meier survival plot 4 weeks after injection indicates that the mice injected with the CAV1 cells survived for a significantly longer period of time than the controls (*P*<0.01); 4I, all xenograft tumors were removed from the experimental mice.

To investigate the function of CAV1 in HCC progression, we first determined the CAV1 levels in metastatic HCC cell lines and tissues compared with non-metastatic ones. By using Q-PCR (Fig. 1A) and western blotting (Fig. 1B), the expression levels of CAV1 in12 HCC cell lines were determined. Metastatic HCC cell lines such as MHCC97-L, MHCC97-H, HCCLM3, HCCLM6, SNU449 and SNU475 express much higher CAV1 expression than that in L02, QGY7701, QGY7703, HepG2, Hep3B and SNU398, while the lowest level of CAV1 among the 12 HCC cells tested was in HepG2 cells. These results provide evidence that CAV1 is associated with the metastatic phenotype of HCC cells. Therefore, HepG2 was selected late on for constructing stable overexpression cells, while MHCC97-H and HCCLM3 were selected and used in RNAi experiments.

In order to determine the expression level of CAV1 in HCC tissues, we performed TMA analysis then. The TMA was constructed from a total of 96 HCC cases (Table 1), consisting of 48 liver cancer tissues each from patients with and without metastasis. TMA analysis showed that HCC tissues with metastasis had higher CAV1 expression levels than those without metastasis (p<0.05, Fig. 1C and 1D, Table 1). This tendency in HCC tissues confirms likely the expression pattern observed in the cell lines.

**Table 1:** Relationship between CAV1 expression and clinicopathological variables in the 96 HCC patients

			CAV-1		
Parameters	Variable	No. of patients	(+)	%	p
Gender	Male	88	28	32	>0.05
	Female	8	4	50	
Age	<65 year	85	45	53	>0.05
	>65 year	11	9	82	
Tumor size	<5 cm	43	23	53	>0.05
	>5 cm	53	30	57	
TNM stage	I-II	66	32	48	>0.05
	III-IV	30	15	50	
Metastasis	No	48	25	52	<0.05★
	Yes	48	38	79	

★ Statistically significant.

Based on the CAV1 expression pattern in HCC-derived cell lines, we stably transfected adenoviral vectors containing CAV1 into HepG2 cells. Stable pools were established (Fig. 1E, F) and used for measuring cell characteristics. For localization of CAV-1 before and after the transfection procedures, we carried out immunofluorescence staining experiments and found that CAV1 is highly enriched in the cytoplasm (Figure S1 in File S1). CAV1 overexpression suppressed the poly-HEMA induced apoptosis of these cells relative to cells transfected with empty pBABE vector (P<0.01; Fig.1G), where it has no effect on cell proliferation and cell cycle (data not shown). Furthermore, we subcutaneously injected HepG2-CAV1 cells into nude mice in order to assess their tumorigenicity. The results showed that CAV1 overexpression can promote the occurrence of visible tumors. Indeed, in our experiments the tumors formed in cells of overexpressing CAV1 were significantly earlier than those of tumors formed from control cells transfected with empty vector. As shown in Fig. 1H, mice injected with CAV1 cells survived for longer than control. The average tumor size and weight were similar between the two group mice (Fig. 1I).

### CAV1 overexpression in non-metastatic cells enables invasion *in vivo*, whereas CAV1 knockdown in metastatic cells inhibits metastasis *in vivo*

**Figure 2:**
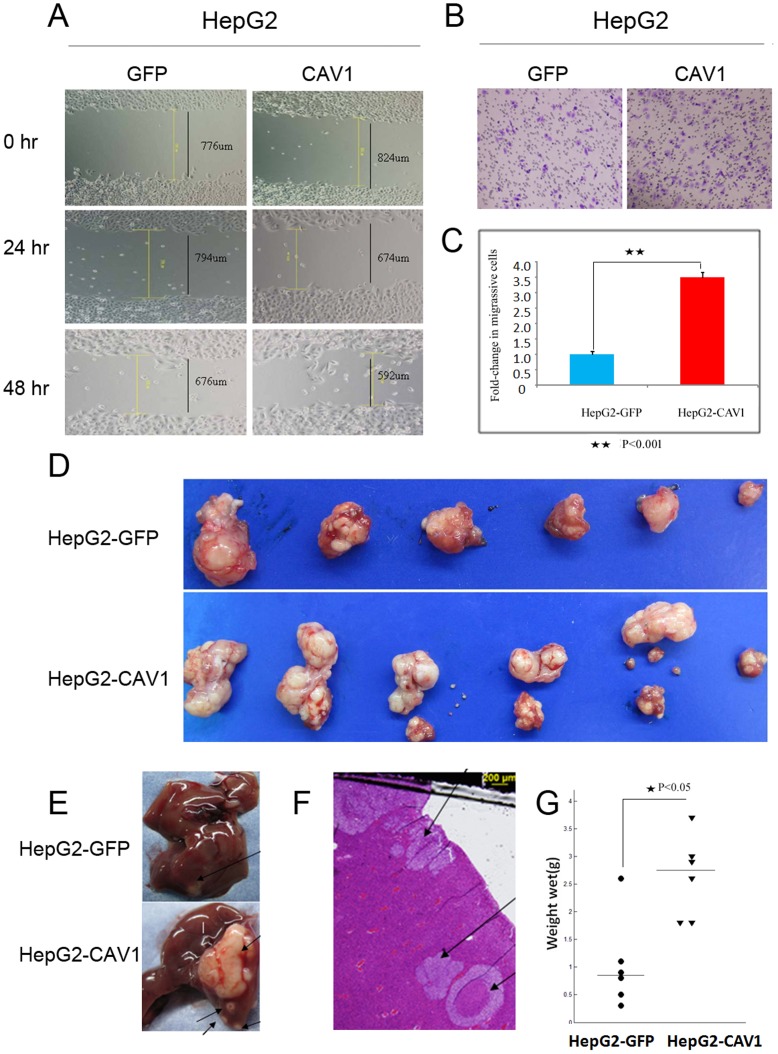
CAV1 modulates HCC cell migration, growth, and metastasis. A, CAV1 overexpression promotes cell migration in HepG2 cells that express GFP and CAV1 using a wound-healing assay. B, Transwell migration assay of HepG2 cell, viewed at ×200 and stained with purple for three independent experiments. C, Comparison analysis with data as *mean* ± *SD*, and the statistically significant differences with Student's t-test. D and E, Effect of CAV1 overexpression on the HepG2 cell metastasis using letivirus–CAV1 via orthotopic implantation. Locoregional metastases, classified as abdominal wall metastases and intrahepatic metastases (black arrow) were observed by day 40. F, representative images show intrahepatic metastases (black arrow) and H&E-stained sections. G, The quantification of average weight of xenograft tumors at 6 weeks is shown. * P<0.05.

Above observations that CAV1 was overexpressed in metastatic live cell lines and tissues suggested the promoting role of CAV1 in HCC metastasis. The wound-healing assay (Fig. 2A) and the Transwell experiments (Fig. 2B and 2C) revealed that CAV1 overexpression can enhance non-metastatic HepG2 cell migration. CAV1 overexpression HepG2 stable clones were then injected subcutaneously into six athymic mice to assess tumorigenicity. By the end of four weeks, no difference in the proliferation could be observed (data not shown). Then, two envelope intact HCC tumors from control or CAV1 overexpression HepG2 stable clones were implanted respectively into the liver of six athymic recipient mice in vivo. Widespread loco regional effects were observed by day 40 (Fig. 2D and 2E), with three mice exhibiting abdominal wall metastases and the other three intrahepatic metastases. The presence of intrahepatic metastatic tumors in mice was confirmed by histologic analysis (Fig. 2F). Distant lung metastasis was not observed. Furthermore, the tumors in the mice injected with CAV1-expressing cells were significantly larger than those injected with control cells (*P*<0.001; Fig. 2D and2G).

**Figure 3:**
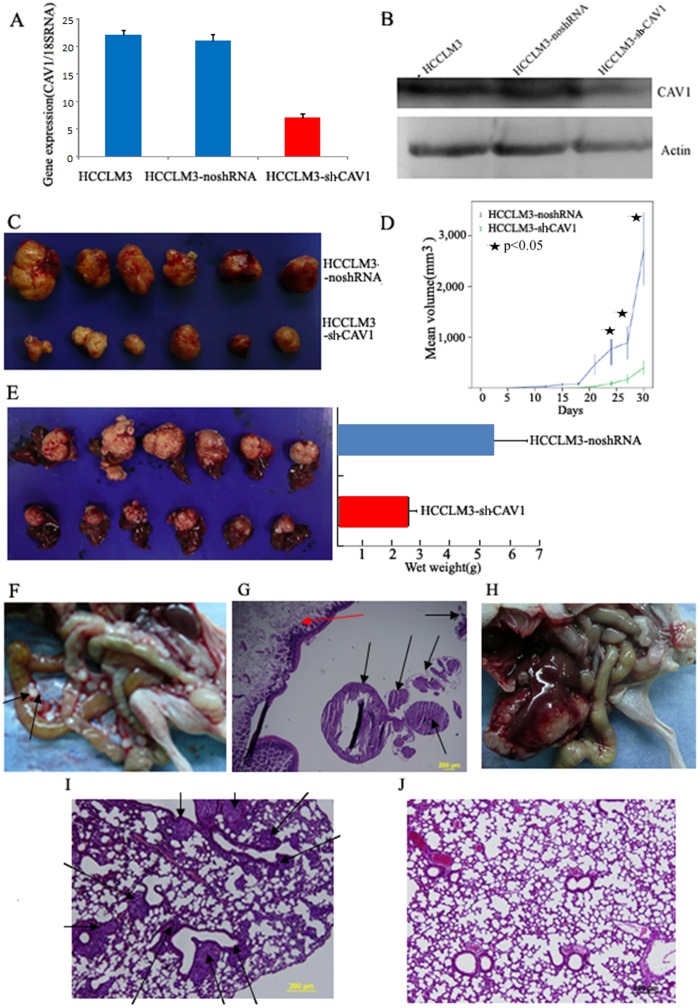
CAV1 knockdown inhibited HCC tumor growth and metastasis. A and B, CAV1 knockdown in MHCCLM3 cells by shRNA-CAV1-1, as demonstrated by qPCR and western blot analysis, with noshRNA as the control. C and D, The growth of subcutaneous tumors was recorded for 30 days, monitored in terms of tumor diameter after 3 days (*mean* ± *SD*), and photographed as mice killed. Cells (5×10^6^) in 0.2 mL of PBS were injected s.c. into right upper flank region of each of the six nude mice. E, After orthotopic implantation, tumor growth was assayed using small piece of subcutaneous tumor. The mice were killed after 6 weeks, and autopsied for photo (left panel) and weight (right panel, *mean* ± *SD*). F, Abdominal metastases to mesenteric lymph nodes (black arrow) in noshRNA tumors. G, Representative images of liver cancer metastasis (black arrow) on the mesenteric fold (red arrow, intestine) (H&E-stain, original magnification, ×40). H, No abdominal metastasis was observed in shRNA-CAV1-1 tumors. I and J, Representative images of metastases formed in lungs of each nude mouse (*n* = 6) at 6 weeks after orthotopic implantation with MHCCLM3 no-shRNA or shRNA-CAV1-1 tumors (black arrow, metastatic tumors).

Results also show that the CAV1 shRNA can efficiently knock down the endogenous CAV1 expression in highly-metastatic MHCCLM3 cells, as indicated by the qPCR and western blot analysis (Fig. 3A and 3B). MHCCLM3 cells (5×10^6^ cells) from each of the stable subclones were injected subcutaneously into six athymic mice to assess tumorigenicity. By the end of four weeks, the shRNA-free tumors grew to an average size of 2,747.7 mm^3^ whereas the shRNA-CAV1-1 403.6 mm^3^, being an approximately 65% inhibition in tumor growth statistically (Fig. 3C and 3D). Furthermore, when small s.c. CAV1-1 shRNA tumor tissues were implanted into the liver of new recipient mice, the tumors were significantly smaller than the tumors in the control mice after 6 weeks (*p*<0.01; Fig. 3E).

We have checked the effects of CAV1 knockdown in MHCCLM3 on long-distance tumor metastasis *in vivo*. Widespread locoregional and distant metastases were observed in the six mice that were orthotopically implanted with MHCCLM3-noshRNA tumors (Fig. 3F). Locoregional metastasis to the abdominal wall occurred in 100% of the cases, 67% metastasized intrahepatically, and 19% metastasized to the abdominal cavity and involved the mesenteric lymph nodes (Fig. 3F and 3G). Lung metastases were observed, and the hematoxylin and eosin (H&E)-stained tissue block sections showed numerous massive metastases in the lung parenchyma (Fig. 3I). However, bare- or tiny-visible locoregional metastasis or metastatic tumors were found in mice lungs that were orthotopically implanted with the MHCCLM3 cells of CAV1 knockdown tumor (Fig. 3H and 3J).

Furthermore, we also verified that CAV1 knockdown not only can inhibit xenograft tumor growth and metastasis in HCCLM3 cells (Fig. 3), but it had the same effect in the other HCC lines, such as MHCC97-H (Figure S2 in File S1). These data suggest that CAV1 downregulation plays a crucial role in the malignant aggression and metastasis of aggressive HCC cells.

### CAV1 overexpression can activate Wnt/β-catenin pathway and induce EMT process

**Figure 4:**
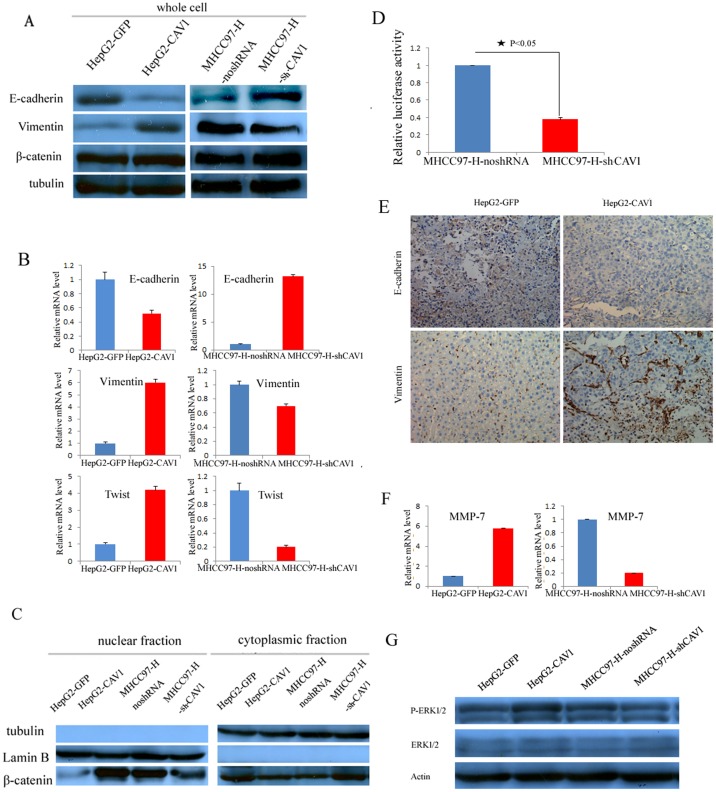
CAV1 changes the subcellular distribution and signaling of β-catenin in EMT. A, CAV1 induce EMT in HCC cells. Whole cell lysates from the control, CAV1-expressing, and sh-CAV1 HCC cells were separated and probed with antibodies for epithelial and mesenchymal markers as indicated. B, CAV1 decreases E-cadherin mRNA level, increases Vimentin and Twist mRNA level. C, The β-catenin distribution in CAV1-expressing and sh-CAV1 HCC cells was determined. The purity of nuclear and cytoplasmic fractions was confirmed by immunoblotting. D, The control and CAV1-RNAi MHCC97-H cells were transiently transfected with TOP-tk to determine the transcriptional activity of β-catenin–mediated signaling. The activity levels are represented as fold activation compared with the control in triplicate wells. E, Immunohistochemistry staining of E-cadherin and Vimentin in the tumors from control and CAV1-overexpression HepG2 cells. F, mRNA level of MMP-7 was determined by Q-PCR. G, Protein levels of phospho-ERK 1/2, and total ERK1/2 were analyzed by western blot. Actin is a loading control.

We have proved that the expression of Epithelial-Mesenchymal Transition (EMT)-markers can be regulated by CAV1 likely through Wnt/β-catenin pathway. The CAV1-overexpression HepG2 cells exhibited downregulation of E-cadherin, a epithelial-marker, whereas the CAV1-knockdown clones showed an upregulation of E-cadherin (Fig. 4A). Vimetin, the mesenchymal marker in EMT, can be upregulated in CAV1-overexpression HepG2 cells, but was downregulated in CAV1-knockdown MHCC97-H cells (Fig. 4A). The corresponding mRNA levels of E-cadherin and Vimetin, were examined respectively by qPCR and showed the same results (Fig. 4B). In addition, the CAV1-overexpression HepG2 cells has high mRNA level of Twist, a transcription factor highly expressed and crucial in promotion of EMT and consequently tumor cell invasion as well [11], while the CAV1-knockdown MHCC97-H cells resulted in a decrease in Twist mRNA level (Fig. 4B).

E-cadherin is a main binding partner of β-catenin and plays an important role in regulating its nuclear localization. The translocation of β-catenin can be investigated by measuring the protein level in the nuclear and cytosolic fractions. We have verified that the CAV1-overexpressed HepG2 cells displayed nuclear accumulation of β-catenin (Fig. 4C). In contrast, markedly increased cytosolic β-catenin and decreased nuclear β-catenin are induced by CAV1 depletion in MHCC97-H (Fig. 4C). The subcellular relocalization of β-catenin can subsequently regulate the transcriptional activities of the Tcf/Lef DNA-binding factors [12], [13]. We have found that reduced nuclear localization of β-catenin in the CAV1-depletion clones is associated with decreased β-catenin transcriptional activity, as detected with the responsive reporter plasmid of β-catenin-Tcf/Lef transfected into cells (Fig. 4D).

Metastasis is an in vivo process and in vitro data showing differential expression of EMT markers is not sufficient. Then we investigated the CAV1-dependent expression of E-cadherin and Vimentin by immunohistochemical staining in the tumors in vivo. The immunohistochemistry results showed that the tumors from CAV1-overexpression HepG2 cells exhibited downregulation of E-cadherin, an epithelial-marker, whereas Vimetin, the mesenchymal marker in EMT, can be upregulated in CAV1-overexpression HepG2 cells. The data have been presented in Fig 4E. We have tried to study the mechanism of how the signal is transduced to the nucleus. It was shown that membrane-type-1 matrix metalloproteinase (MT1-MMP) co-localizes with CAV1 at the perinuclear region and it may be translocated to the nucleus via caveolae mediated endocytosis in HCC [14]. Data has also shown that CAV1 is needed in epidermal growth factor (EGF)-induced mouse embryonic stem cell migration, extracellular signal-regulated protein kinase (ERK) phosphorylation and MMP-2 expression [15]. However, CAV1 has been reported as a negative regulator of MMP-1 gene expression in human dermal fibroblasts via inhibition of ERK signaling pathway [16]. Han et al has shown that CAV1 gene could inhibit pancreatic carcinoma cell invasion, at least in part, probably through ERK-MMP signal pathway [17]. Since EMT is always dependent on the activation of ERK signaling pathways [18], so we investigated the effect of CAV1 in ERK phosphorylation level and MMPs mRNA expression in HCC metastatic process.

We found MMP-7 was increased in CAV1 expressing HepG2 cells (Fig 4F), while MMP-1 and MMP-2 mRNA level have no significant change (data not shown). For activity of ERK examined by Western blot, we observed that overexpression of CAV1 induced activation of ERK in CAV1 overexpressing HepG2 cells compared with those of control cells (Fig 4G).

## Discussion

CAV1 has been implicated in the tumorogenesis. It was shown that CAV1 expression difference is tumor cells type-dependent. For instance, while CAV1 downregulation is typical for ovarian, lung, and mammary carcinomas, it is upregulated in bladder, thyroid and prostate carcinomas [2]. Furthermore, CAV1 contributes to metastatic phenotype in different types of carcinomas [19], [20]. De-regulation of CAV1 expression in HCC tissues has been documented [3]. Yokomori et al. reported that CAV1 expression elevated in cirrhotic liver and HCC, while it was almost undetectable in normal liver [21]. On the other hand, it was shown that CAV1 expression was inactivated in HCC cell lines by aberrant methylation [22]. These observations do not provide clear evidence about the role of CAV1 in hepatocellular carcinogenesis, yet the function of CAV1 in HCC metastasis still remain poorly understood.

Our observation in human HCC samples was in accordance with previous reports. CAV1 was absent in non-metastatic liver cell lines but was exclusively expressed in metastatic cell lines. Together with the significant overexpression in metastatic liver tissues, it is conceivable that CAV1 positively regulates HCC progression and metastasis. Cell migration and invasion are critical events in metastasis which involve the relocation of signaling molecules that establish the polarity and induce dynamic cytoskeletal changes of the cell. CAV1 overexpressing and knockdown stable clones were established in HCC cells and their *in vitro* cellular effects and *in vivo* tumourigenicity and metastatic potential were examined. Overexpression of CAV1 promoted liver cancer cell motility and invasiveness *in vitro*, as well as metastasis *in vivo*. Conversely, knockdown of CAV1 in metastatic HCC cells markedly inhibited the tumour metastatic potential *in vivo*. In summary, our results have shown the exclusive expression of CAV1 in metastatic HCC cell lines and clinical samples. The definitive role of CAV1 promoting HCC metastasis was demonstrated.

EMT and related pathways have been studied in metastatic HCCs, to investigate whether EMT is a crucial step in the conversion of early stage tumors into invasive malignancies [13], [23]. Sun reported that Twist1 (an EMT trigger marker) was frequently overexpressed in the nuclear relocation occurring in VM (vasculogenic mimicry)-positive HCCs, and Twist1 nuclear expression in EMT was likewise associated with VM formation, and with the tumor invasion and metastasis of HCC [24]. Yang discovered that a comprehensive profile of EMT markers in HCC, and the independent and collaborative effects of Snail (the other trigger marker) and Twist on HCC metastasis were confirmed through different assays [25].

β-catenin signaling pathways is important in metastatic HCC. Liu has proven that hypoxia could induce β-catenin overexpression and/or intracellular accumulation in four HCC cell lines through downregulating the endogenous degradation machinery, and hypoxia can also promote an invasion *in vitro* and metastasis *in vivo* for MHCC97 and Hep3B cells [26]. Hypoxic MHCC97 and Hep3B cells exhibited molecular alterations consistent with EMT, characterized by the loss of epithelial cell markers and up-regulation of mesenchymal markers, as well as the increase of MMP2. However, silencing of β-catenin in these hypoxic cells reversed EMT and repressed metastatic potential.

Preventing caveolae assembly through CAV1 reducing results in E-cadherin accumulation at cell borders and the formation of tightly adherent cells [27]. Accordingly, the nuclear translocation of β-catenin occurred after E-cadherin down-regulation, and Lef transcription was activated after β-catenin translocated into the nucleus, where the Tcf/Lef transcript can be activated [28]. We also prove evidence that the CAV1 up-regulation induced E-cadherin downregulation. Consequently, CAV1 stimulation can induce an activation of the Wnt/β-catenin-Tcf/Lef pathway, especially at the transcriptional level.

Due to the crucial roles of CAV1 and MMPs in regulating tumor progression and metastasis, a growing number of studies have investigated a possible relationship between these molecules. Published data suggests that CAV1 can be an important regulator of the expression and activity of MMPs, including CAV1 unregulated MMP-2 and 9 in melanoma cells [29]. Various signaling pathways have been shown to induce MMPs gene expression, including ERK1/2, JNK and p38 MAPK et al [30]–[31]. In this study, we have investigated the specific contribution of CAV1 to MMPs gene expression in human HCC cell lines. Additionally we show that CAV1 increases MMP-7 expression via activation of ERK1/2 signaling pathways.

Taken together, these results suggest that CAV1 induces the HCC cell invasion and metastasis partial by enhancing MMP-7 expression, associated with decreased E-cadherin expression, resulting in EMT and acting through ERK activation.

## Supporting Information

File S1 **Supporting information.** Figure S1. Subcellular localization of CAV1 was examined by immunofluorescence microscopy in HepG2 cells. Figure S2. CAV1 knockdown inhibit HCC tumor growth and metastasis in MHCC97H cells. A, B, shRNA-CAV1-1was used to knockdown CAV1 in MHCC97H cells, as demonstrated by real time RT-PCR and western blotting, where noshRNA was used as control. C, D, Cells (5×10^6^) in 0.2 mL PBS were injected s.c. into the right upper flank region of each of 6 nude mice. The growth of subcutaneous tumor was recorded for 30 days, tumor growth was monitored for 3 days by measuring the tumor diameters (*mean ± SD*) then the mice were killed and tumors were removed. E, orthotopic implantation was assayed using small piece of subcutaneous tumor. The mice were observed for 6 weeks, killed, and autopsied. Tumors were weighed at 6 weeks; the weight is indicated (*mean ± SD*). F, G, Representative images of metastases that formed in lungs of each nude mouse (*n* = 6) at 6 weeks after orthotopicly implanted with MHCC97H no-shRNA or shRNA-CAV1-1 tumors. Black arrows indicate metastatic tumors in lung. Original magnification, ×100.
(DOC)

## References

[1] Gupta GP, Massague J (2006) Cancer metastasis: building a framework. Cell 127: 679–695. 17110329 10.1016/j.cell.2006.11.001

[2] Williams TM, Lisanti MP (2005) Caveolin-1 in oncogenic transformation, cancer, and metastasis. Am J Physiol Cell Physiol 288: C494–506. 15692148 10.1152/ajpcell.00458.2004

[3] Liu P, Rudick M, Anderson RG (2002) Multiple functions of caveolin-1. J Biol Chem 277: 41295–41298. 12189159 10.1074/jbc.R200020200

[4] Cokakli M, Erdal E, Nart D, Yilmaz F, Sagol O, et al (2009) Differential expression of Caveolin-1 in hepatocellular carcinoma: correlation with differentiation state, motility and invasion. BMC Cancer 9: 65. 19239691 10.1186/1471-2407-9-65 PMC2656543

[5] Tang Y, Zeng X, He F, Liao Y, Qian N, et al (2012) Caveolin-1 is related to invasion, survival, and poor prognosis in hepatocellular cancer. Med Oncol 29: 977–984. 21416157 10.1007/s12032-011-9900-5

[6] Zhang ZB, Cai L, Zheng SG, Xiong Y, Dong JH (2009) Overexpression of caveolin-1 in hepatocellular carcinoma with metastasis and worse prognosis: correlation with vascular endothelial growth factor, microvessel density and unpaired artery. Pathol Oncol Res 15: 495–502. 19434519 10.1007/s12253-008-9144-7

[7] Choi HN, Kim KR, Park HS, Jang KY, Kang MJ, et al (2007) Expression of caveolin in hepatocellular carcinoma: association with unpaired artery formation and radiologic findings. Korean J Hepatol 13: 396–408. 17898556 10.3350/kjhep.2007.13.3.396

[8] Yan J, Lu Q, Dong J, Li X, Ma K, et al (2012) Hepatitis B virus X protein suppresses caveolin-1 expression in hepatocellular carcinoma by regulating DNA methylation. BMC Cancer 12: 353. 22894556 10.1186/1471-2407-12-353 PMC3522558

[9] Yang SF, Yang JY, Huang CH, Wang SN, Lu CP, et al (2010) Increased caveolin-1 expression associated with prolonged overall survival rate in hepatocellular carcinoma. Pathology 42: 438–445. 20632820 10.3109/00313025.2010.494293

[10] Trimmer C, Whitaker-Menezes D, Bonuccelli G, Milliman JN, Daumer KM, et al (2010) CAV1 inhibits metastatic potential in melanomas through suppression of the integrin/Src/FAK signaling pathway. Cancer Res 70: 7489–7499. 20709760 10.1158/0008-5472.CAN-10-0900 PMC2948597

[11] Kang Y, Massague J (2004) Epithelial-mesenchymal transitions: twist in development and metastasis. Cell 118: 277–279. 15294153 10.1016/j.cell.2004.07.011

[12] Stockinger A, Eger A, Wolf J, Beug H, Foisner R (2001) E-cadherin regulates cell growth by modulating proliferation-dependent beta-catenin transcriptional activity. J Cell Biol 154: 1185–1196. 11564756 10.1083/jcb.200104036 PMC2150811

[13] Conacci-Sorrell M, Simcha I, Ben-Yedidia T, Blechman J, Savagner P, et al (2003) Autoregulation of E-cadherin expression by cadherin-cadherin interactions: the roles of beta-catenin signaling, Slug, and MAPK. J Cell Biol 163: 847–857. 14623871 10.1083/jcb.200308162 PMC2173691

[14] Ip YC, Cheung ST, Fan ST (2007) Atypical localization of Membrane Type 1-Matrix Metalloproteinase in the nucleus is associated with aggressive features of hepatocellular carcinoma. Mol Carinog 46: 225–230. 10.1002/mc.20270 17219425

[15] Park JH, Han HJ (2009) Caveolin-1 plays important role in EGF-induced migration and proliferation of mouse embryonic stem cells: involvement of PI3K/Akt and ERK. Am J Physiol Cell Physiol 297: C935–C944. 19625610 10.1152/ajpcell.00121.2009

[16] Haines P, Samuel GH, Cohen H, Trojanowska M, Bujor AM (2011) Caveolin-1 is a negative regulator of MMP-1 gene expression in human dermal fibroblasts via inhibition of Erk1/2/Ets1 signaling pathway. J Dermatol Sci 64: 210–6. 21925842 10.1016/j.jdermsci.2011.08.005 PMC3826600

[17] Han F1, Zhu HG (2010) Caveolin-1 regulating the invasion and expression of matrix metalloproteinase (MMPs) in pancreatic carcinomacells. J Surg Res 159: 443–50. 20031158 10.1016/j.jss.2009.03.079

[18] Lin ZH, Wang L, Zhang JB, Liu Y, Li XQ, Guo L, Zhang B, Zhu WW, Ye QH (2014) MST4 promotes hepatocellular carcinoma epithelial-mesenchymal transition and metastasis via activation of the p-ERK pathway. Int J Oncol 45: 629–640. 24859810 10.3892/ijo.2014.2455

[19] Ho CC, Huang PH, Huang HY, Chen YH, Yang PC, et al (2002) Up-regulated caveolin-1 accentuates the metastasis capability of lung adenocarcinoma by inducing filopodia formation. Am J Pathol 161: 1647–1656. 12414512 10.1016/S0002-9440(10)64442-2 PMC1850800

[20] Lu Z, Ghosh S, Wang Z, Hunter T (2003) Downregulation of caveolin-1 function by EGF leads to the loss of E-cadherin, increased transcriptional activity of beta-catenin, and enhanced tumor cell invasion. Cancer Cell 4: 499–515. 14706341 10.1016/s1535-6108(03)00304-0

[21] Yokomori H, Oda M, Yoshimura K, Nomura M, Wakabayashi G, et al (2003) Elevated expression of caveolin-1 at protein and mRNA level in human cirrhotic liver: relation with nitric oxide. J Gastroenterol 38: 854–860. 14564631 10.1007/s00535-003-1161-4

[22] Hirasawa Y, Arai M, Imazeki F, Tada M, Mikata R, et al (2006) Methylation status of genes upregulated by demethylating agent 5-aza-2'-deoxycytidine in hepatocellular carcinoma. Oncology 71: 77–85. 17341888 10.1159/000100475

[23] Garber K (2008) Epithelial-to-mesenchymal transition is important to metastasis, but questions remain. J Natl Cancer Inst 100: : 232–233, 239. 10.1093/jnci/djn032 18270330

[24] Sun T, Zhao N, Zhao XL, Gu Q, Zhang SW, et al (2010) Expression and functional significance of Twist1 in hepatocellular carcinoma: its role in vasculogenic mimicry. Hepatology 51: 545–556. 19957372 10.1002/hep.23311

[25] Yang MH, Chen CL, Chau GY, Chiou SH, Su CW, et al (2009) Comprehensive analysis of the independent effect of twist and snail in promoting metastasis of hepatocellular carcinoma. Hepatology 50: 1464–1474. 19821482 10.1002/hep.23221

[26] Liu L, Zhu XD, Wang WQ, Shen Y, Qin Y, et al (2010) Activation of beta-catenin by hypoxia in hepatocellular carcinoma contributes to enhanced metastatic potential and poor prognosis. Clin Cancer Res 16: 2740–2750. 20460486 10.1158/1078-0432.CCR-09-2610

[27] Orlichenko L, Weller SG, Cao H, Krueger EW, Awoniyi M, et al (2009) Caveolae mediate growth factor-induced disassembly of adherens junctions to support tumor cell dissociation. Mol Biol Cell 20: 4140–4152. 19641024 10.1091/mbc.E08-10-1043 PMC2754928

[28] Vincan E, Barker N (2008) The upstream components of the Wnt signalling pathway in the dynamic EMT and MET associated with colorectal cancer progression. Clin Exp Metastasis 25: 657–663. 18350253 10.1007/s10585-008-9156-4

[29] Felicetti F, Parolini I, Bottero L, Fecchi K, Errico MC, Raggi C, et al (2009) Caveolin-1 Tumor - promoting role in human melanoma. Int J Cancer 125: 1514–1522. 19521982 10.1002/ijc.24451 PMC2805039

[30] Sternlicht MD, Werb Z (2001) How matrix metalloproteinases regulate cell behavior. Annu Rev Cell Dev Biol 17: 463–516. 11687497 10.1146/annurev.cellbio.17.1.463 PMC2792593

[31] Vincenti MP, Brinckerhoff CE (2002) Transcriptional regulation of collagenase (MMP-1, MMP-13) genes in arthritis: integration of complex signaling pathways for the recruitment of gene-specific transcription factors. Arthritis Res 4: 157–164. 12010565 10.1186/ar401 PMC128926

