# Supplementary material for: CAV1 Promotes HCC Cell Progression and Metastasis through Wnt/β-Catenin Pathway
Source: PLoS One. 2014 Sep 2;9(9):e106451. doi: 10.1371/journal.pone.0106451 (PMC4152279; doi:10.1371/journal.pone.0106451)
Supplement: File S1 — Supporting information. Figure S1. Subcellular localization of CAV1 was examined by immunofluorescence microscopy in HepG2 cells. Figure S2. CAV1 knockdown inhibit HCC tumor growth and metastasis in MHCC97H cells. A, B, shRNA-CAV1-1was used to knockdown CAV1 in MHCC97H cells, as demonstrated by real time RT-PCR and western blotting, where noshRNA was used as control. C, D, Cells (5×10^6^) in 0.2 mL PBS were injected s.c. into the right upper flank region of each of 6 nude mice. The growth of subcutaneous tumor was recorded for 30 days, tumor growth was monitored for 3 days by measuring the tumor diameters (mean ± SD) then the mice were killed and tumors were removed. E, orthotopic implantation was assayed using small piece of subcutaneous tumor. The mice were observed for 6 weeks, killed, and autopsied. Tumors were weighed at 6 weeks; the weight is indicated (mean ± SD). F, G, Representative images of metastases that formed in lungs of each nude mouse (n = 6) at 6 weeks after orthotopicly implanted with MHCC97H no-shRNA or shRNA-CAV1-1 tumors. Black arrows indicate metastatic tumors in lung. Original magnification, ×100. (DOC) [file pone.0106451.s001.doc]

**SUPPLEMENTAL INFORMATION**

**Supplemental Experimental Procedures**

**Cell Line Establishment and Dataset Collection**

For in-depth study of the underlying mechanisms of HCC metastasis, Li and Tang et al. in the Liver Cancer Institute, Zhongshan Hospital, Fudan University has established a “stepwise metastatic human hepatocellular carcinoma (HCC) cell lines”, named as MHCC97-L (97L), MHCC97-H (97H), HCCLM3 (LM3), HCCLM6 (LM6) with increased metastatic potential in general [1].

The other three HCC cell lines, SNU398, SNU449, and SNU475, were developed from Korea patient. SNU449 and SNU475 are those with invasive potential, but SNU398 has no invasive potential [2,3]. Hep3B cell line (non-metastatic) was established from a patient of 8-year-old black boy [4]. For comparisons, we have also used another set of HCC cell lines, namely L02, QGY7701, QGY7703, Huh7, and HepG2 (these 5 ones used as ordinary HCCs).

**Cell Migration Assay**

For transwell cell migration assays, a total of 1×10^5^ cells was then suspended in 100μL DMEM containing 0.1% FBS and added to the upper chamber of 24 wells (CytoSelect)., while 600μL DMEM containing 10% FBS was placed in the lower chamber as the chemoattractant. After 24h incubation at 37℃ in 5% CO_2_, the cells that remained on the upper surface of the membrane were removed by wiping with a cotton swabs. Migrated cells attached to the lower surface of the membrane were fixed with 4% paraformaldehyde and stained with 0.5% crystal violet. Cells were counted and photographed (magnification, ×100) for ten random microscopic fields per membrane, and averaged. Three independent experiments were performed in triplicates.

**Wound-healing Assay**

A wound was made according to CytoSelect™ 24-Well Wound Healing Assay protocol. Briefly, add 500µL of cell suspension containing 2.5～5.0×10^5^ cells to each well by carefully inserting the pipet tip through the open end at the top of the insert. For optimal cell dispersion, add 250µL of cell suspension to either side of the open ends at the top of the insert. Incubate cells in a cell culture incubator overnight or until a monolayer forms. Carefully remove the insert from the well to begin the wound healing assay. The phase contrast images of the wounds were recorded at 37℃for incubations of 0, 24, 48 hours. Visualize wells under a light microscope. Three separate experiments were performed.

**Anoikis Assay**

Six-well plates were treated with Poly-2-hydroxyethylmethacrylate (poly-HEMA) (Sigma) following the Folkman and Moscona method[5]­. Briefly, wells were treated with a 1mL solution of poly-HEMA diluted in ethanol 95% (12mg/mL) and left to dry at room temperature. Before use, poly-HEMA-coated dishes were washed twice in phosphate-buffered solution (PBS) and once in Hank's solution. Seeded were 106cells/well in DMDM 1% FBS and incubated at 37℃ in 5% CO2 atmosphere. Viable and dead cells were counted after 24 hours. Detection of apoptotic cells were obtained by flow cytometric analysis (Epics Altra, Beckman Coulter) of annexin V-labelled cells performed according to the manufacturer's instructions (Roche). Briefly, 1×10^6^ cells were washed with PBS and incubated with 100μL of the following solution: 20μL of annexin V-FITC, 20μL of propidium iodide (PI), and 1mL of PBS buffer. PI incorporation was evaluated in association with the fluorescent signal intensity to allow the discrimination of necrotic and apoptotic cells.

**Western Blotting Analysis**

Protein lysates were prepared from transfected stable pool cells in a buffer containing 50mM Tris-HCl (pH 8.0), 150mM NaCl, 0.1% SDS, 0.5% deoxycholate, 1% NP-40, 1mM EDTA, 1mM PMSF, 25mM NaF, and cocktail protease inhibitors (Roche). Protein lysates were resolved by SDS-PAGE, followed by Western blotting analysis using E-cadherin monoclonal antibody (MAb, BioRaD), vimentin MAb (BioRaD), β-catenin antibody (Cell Signaling), tublin antibody (Biyuntian), actin antibody (Genscript) and lamin B antibody (Santa Cruz Biotechnology).

**Reporter Gene Assay**

To determine the endogenous β-catenin/Tcf transcriptional activities, the cells were transfected using Lipofectin 2000 with the reporter construct TOP-tk. Reporter gene assays were determined using the Dual-Luciferase Reporter Assay System (Promega). At 24h after the transfection, the cells were lysed in 100μL passive lysis buffer and the soluble fractions were used for luciferase assays following the manufacturer’s instructions.

**Tumorigenicity *in vivo***

Eighteen nude mice (Bab c nu/nu), ages 4～6 weeks (about 18g of weight) were randomly divided into three groups. A total of 2.5×10^6^ targeted cells, showing either knockdown or overexpression of CAV1, were injected subcutaneously into the right flank of nude mice. Growth curves were plotted using average tumor volume within each experimental group at the set time points. The tumor dimensions were measured every 3 days using a digital caliper, and the tumor volume calculated using the following formula: V=ab^2^/2 in mm3, where a and b are the longest and the shortest perpendicular diameters of the tumor, respectively. Tumor growth was observed for at least 4 weeks. The tumorigenic experiments *in vivo* were performed with 6 mice in each treatment group. Mice handling and experimental procedures followed institutional guidelines.

Figure S1


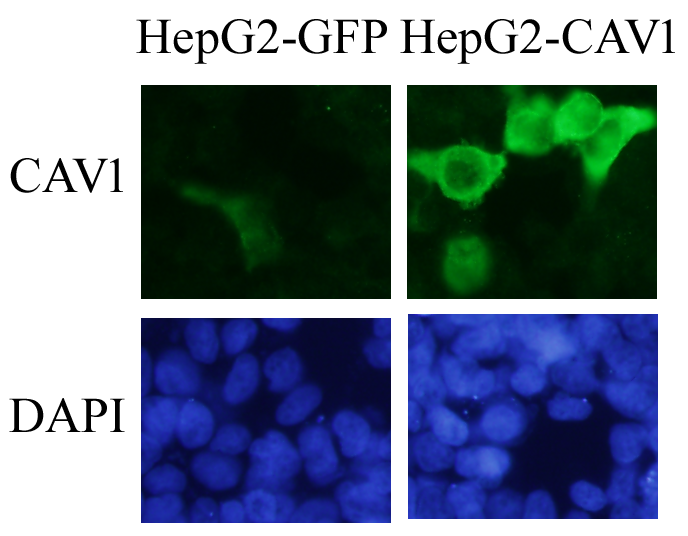


**Figure S2**


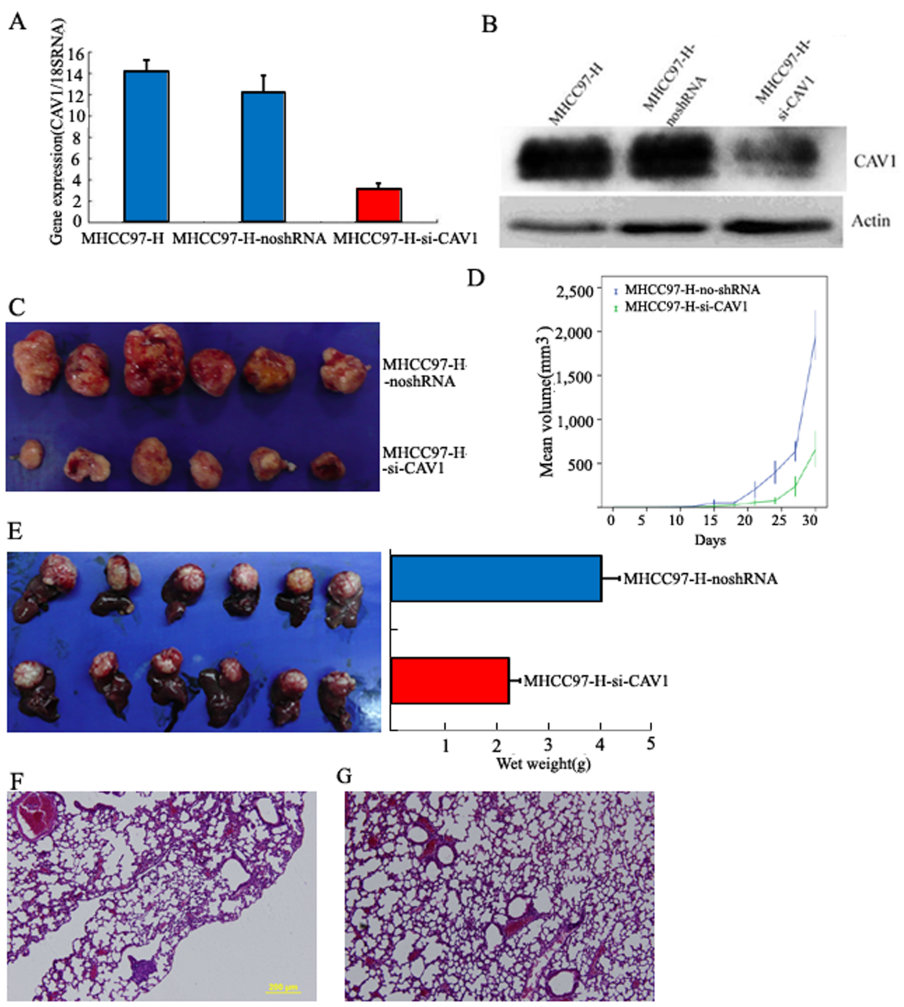


1. Li Y Tian B, Yang J, Zhao L, Wu X, Ye SL, et al. Stepwise metastatic human hepatocellular carcinoma cell model system with multiple metastatic potentials established through consecutive in vivo selection and studies on metastatic characteristics. J Cancer Res Clin Oncol 2004 Aug;130(8):460-468.
2. Park JG, Lee SK, Hong IG, Kim HS, Lim KH, Choe KJ, et al. MDR1 gene expression: its effect on drug resistance to doxorubicin in human hepatocellular carcinoma cell lines. J Natl Cancer Inst 1994 May 4;86(9):700-705.
3. Park JG, Lee JH, Kang MS, Park KJ, Jeon YM, Lee HJ, et al. Characterization of cell lines established from human hepatocellular carcinoma. Int J Cancer 1995 Jul 28;62(3):276-282.
4. Knwles BB, Aden DP. Human hepatoma derived cell line, process for preparation thereof, and uses therefor. US Patent 1983;4(393):5.
5. Folkman J, Moscona A. Role of cell shape in growth control. Nature 1978 Jun 1;273(5661):345-349.
